# Transmission–reflection optoacoustic ultrasound (TROPUS) computed tomography of small animals

**DOI:** 10.1038/s41377-019-0130-5

**Published:** 2019-01-30

**Authors:** Elena Merčep, Joaquín L. Herraiz, Xosé Luís Deán-Ben, Daniel Razansky

**Affiliations:** 1 Faculty of Medicine, Technical University of Munich, Munich, Germany; 2 iThera Medical GmbH, Munich, Germany; 3 Nuclear Physics Group and UPARCOS, Complutense University of Madrid, CEI Moncloa, Madrid, Spain; 4 Health Research Institute of Hospital Clínico San Carlos (IdISSC), Madrid, Spain; 5 Institute for Biological and Medical Imaging (IBMI), Helmholtz Center Munich, Neuherberg, Germany; 6 Faculty of Medicine and Institute of Pharmacology and Toxicology, University of Zurich, Zurich, Switzerland; 7 Institute for Biomedical Engineering and Department of Information Technology and Electrical Engineering, ETH Zurich, Zurich, Switzerland

**Keywords:** Imaging and sensing, Photonic devices

## Abstract

Rapid progress in the development of multispectral optoacoustic tomography techniques has enabled unprecedented insights into biological dynamics and molecular processes in vivo and noninvasively at penetration and spatiotemporal scales not covered by modern optical microscopy methods. Ultrasound imaging provides highly complementary information on elastic and functional tissue properties and further aids in enhancing optoacoustic image quality. We devised the first hybrid transmission–reflection optoacoustic ultrasound (TROPUS) small animal imaging platform that combines optoacoustic tomography with both reflection- and transmission-mode ultrasound computed tomography. The system features full-view cross-sectional tomographic imaging geometry for concomitant noninvasive mapping of the absorbed optical energy, acoustic reflectivity, speed of sound, and acoustic attenuation in whole live mice with submillimeter resolution and unrivaled image quality. Graphics-processing unit (GPU)-based algorithms employing spatial compounding and bent-ray-tracing iterative reconstruction were further developed to attain real-time rendering of ultrasound tomography images in the full-ring acquisition geometry. In vivo mouse imaging experiments revealed fine details on the organ parenchyma, vascularization, tissue reflectivity, density, and stiffness. We further used the speed of sound maps retrieved by the transmission ultrasound tomography to improve optoacoustic reconstructions via two-compartment modeling. The newly developed synergistic multimodal combination offers unmatched capabilities for imaging multiple tissue properties and biomarkers with high resolution, penetration, and contrast.

## Hybridized optoacoustic ultrasound computed tomography

A three-in-one imaging platform combines the advantages of each individual technique to provide whole body tomographic imaging of small animals. Developed by the group of Daniel Razansky from the University of Zurich and ETH Zurich in Switzerland and collaborators in Germany and Spain, the hybrid platform combines optoacoustic tomography with reflection and transmission mode ultrasonography. By launching ultrasound and laser pulses into tissues, the technique allows the construction of cross-sectional tomographic images that reveal fine details on organ function, tissue vascularization, reflectivity, stiffness and density. As an added value of the hybrid combination, images retrieved by one modality are also used to enhance the reconstruction quality of the other two modalities. The platform could thus be used for probing and quantifying multiple anatomical, functional and molecular properties of tissues in health and disease.

## Introduction

Over the last years, tremendous advancements have been introduced into multispectral optoacoustic tomography (MSOT) technology^1,2^. Those have enabled the implementation of ultrafast imaging systems for volumetric visualization of organ dynamics and motion^3,4^, whole body imaging of small animals with unsurpassed image quality^5^, sensitive deep-tissue detection of molecular agents, and disease biomarkers^6–8^. MSOT brings along important advantages in terms of label-free anatomical and functional contrast arising from intrinsic tissue components, such as oxy- and deoxyhemoglobin, melanin, bilirubin, lipids, and water. In particular, the strong optical absorption of hemoglobin allows the visualization of vascular structures and hemodynamic responses, maintaining submillimeter resolutions at depths of several centimeters within highly scattering living tissues in the near-infrared spectrum. The great preclinical potential of MSOT has also encouraged the translation of this technology into the clinics with dedicated handheld^9,10^ and endoscopic^11,12^ probes introduced for high-performance imaging of human subjects.

Ultrasound (US) tissue contrast provides highly complementary information on elastic and functional properties^13^. At present, pulse-echo (reflection-mode) ultrasonography remains the most commonly employed clinical imaging modality^14^. It is equally exploited in preclinical research, representing an essential tool in many active research areas, such as neuroimaging^15^, cardiology^16^, tumor angiogenesis^17^, or the development of novel contrast enhancement approaches^18,19^. Transmission-mode ultrasound-computed tomography (TUCT) can instead map the distribution of speed of sound and acoustic attenuation, which are representative of a different set of physiological tissue properties, such as stiffness, density, and temperature^20,21^. In this regard, TUCT has been used to map the distribution of speed of sound (SoS) and acoustic attenuation (AA) in the female breast^22,23^, where recent clinical trials suggest a superior performance with respect to standard screening approaches in terms of safety, examination time, and patient comfort^24–26^.

The integration of US-based imaging approaches into multimodal optoacoustic ultrasound (OPUS) platforms has previously been shown to complement and enhance advantages of the stand-alone modalities. The hybridization between optoacoustic (OA) and reflection-mode (pulse-echo) US imaging has recently been achieved with linear^27^, concave^28^, or multisegment arrays^29,30^. Multimodal endoscopic^31,32^ and microscopic^33,34^ imaging systems based on single-element transducers have also been suggested. Other efforts have been directed toward enhancing image quality by incorporating complementary information in reconstruction algorithms^35^, clearly evincing added value of the multimodal approach.

Efficient hybridization between the various OA and US imaging modalities is often hampered by the fundamental differences in the underlying contrast mechanisms and image-formation strategies. While reflection-mode US is commonly performed with linear or convex arrays from a single-access point to the sample, optimal transmission-mode US and OA reconstructions are achieved with large tomographic coverage from multiple views around the imaged region. The detected signal intensity ranges can also differ substantially for transmitted versus back-scattered US waves or OA responses^36,37^, which implies different implementations of the front-end signal generation and amplification electronics and digitization chains.

**Fig. 1:**
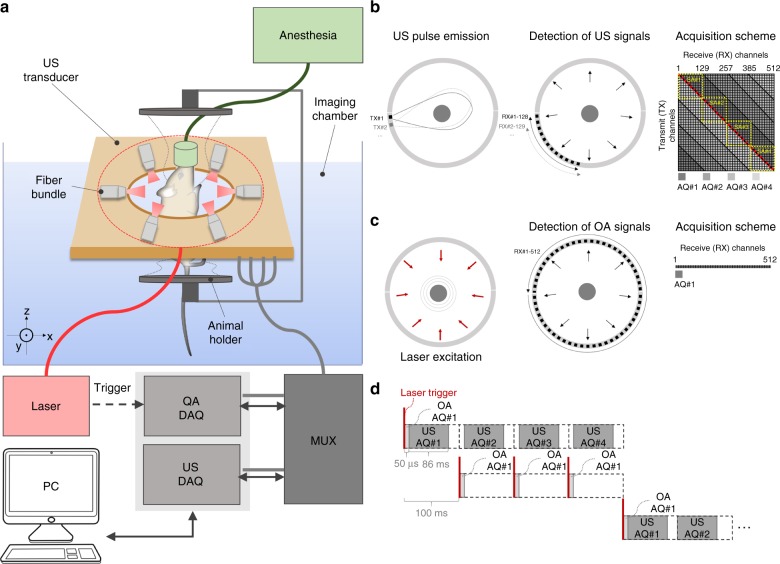
Diagram of the transmission–reflection optoacoustic ultrasound (TROPUS) imaging platform for whole-body computed tomography of small animals. **a** Block diagram illustrating the key components of the experimental prototype. A dedicated digital acquisition and signal generation hardware was developed for synchronizing the excitation and detection of broadband ultrasound signals by a 512-element cylindrically focused full-ring array. **b** Both reflection- and transmission-mode ultrasound are based on sequential active excitation of each array element and parallel detection of 128 signals, i.e., four acquisition cycles were required for collecting signals from all the 512 channels. **c** In the optoacoustic data acquisition mode, signals generated by each laser pulse were recorded by all the 512 channels simultaneously. **d** The multiplexing unit synchronizing the optoacoustic and ultrasound imaging modes was based on the predefined timing scheme triggered with the laser pulses

Here, we devised a hybrid transmission–reflection optoacoustic ultrasound (TROPUS) imaging platform for whole-body computed tomography of small animals (Fig. 1a). The system features full-view cross-sectional tomographic imaging geometry for concomitant noninvasive mapping of the absorbed optical energy, acoustic reflectivity, speed of sound, and acoustic attenuation in whole live mice with submillimeter resolution. For this, a dedicated multiplexer unit was further developed to control and synchronize the excitation and detection of signals by the custom-made full-ring 512-element cylindrically focused US transducer array (see Methods for details of the experimental setup).

## Results

### Multimodality imaging performance characterization

**Fig. 2:**
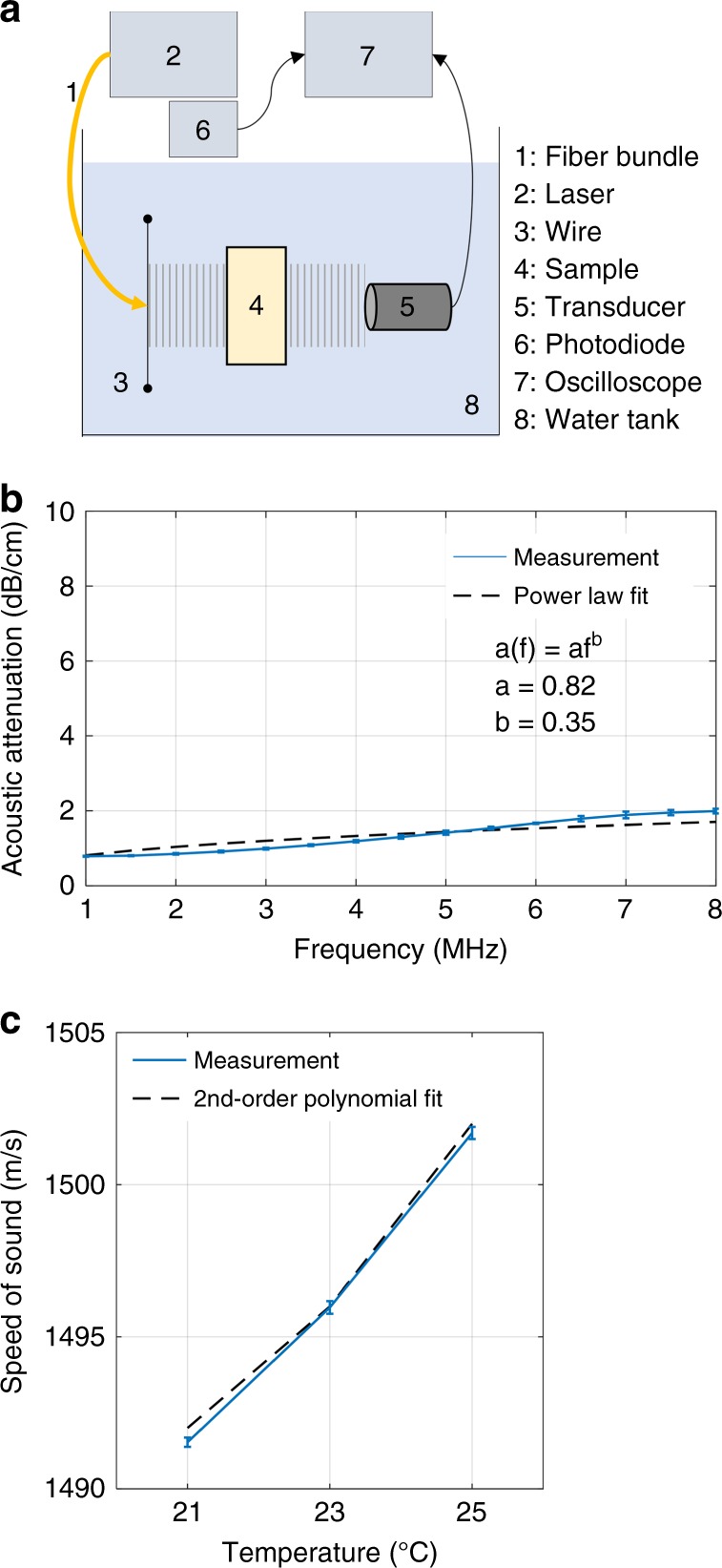
Characterization of accuracy of the acoustic attenuation and speed of sound estimation. **a** Schematics of the acoustic transmission measurement setup. **b** Frequency-dependent acoustic attenuation of the phantom in a range from 1 MHz to 8 MHz. **c** Temperature-dependent speed of sound of the phantom

Performance of the developed system was tested by imaging a tissue-mimicking phantom, whose acoustic properties, namely, attenuation and speed of sound, were first estimated using an acoustic transmission setup with the phantom placed in between a light-absorbing 0.3-mm thick carbon black suture generating ultrasound signals and a single-element transducer (Fig. 2a). The measured and fitted acoustic attenuation as a function of frequency is shown in Fig. 2b, where the coefficients a and b of the power law (Eq. (5)) are estimated to be a = 0.82 and b = 0.35, respectively. Considering the transmit frequency of 6 MHz used in the experiment, this results in the expected acoustic attenuation of the phantom material α = 0.26 dB/MHz/cm. Figure 2c shows the measured and fitted speed of sound as a function of ambient water temperature, where the coefficients of the 2^nd^ order polynomial fit are estimated to be p1 = 0.2, p2 = −4.9, and p3 = 1523.5. Considering the ambient water temperature of 21.3 °C measured during the phantom experiment, the expected speed of sound of the phantom material is thus c = 1492.1 m/s.

**Fig. 3:**
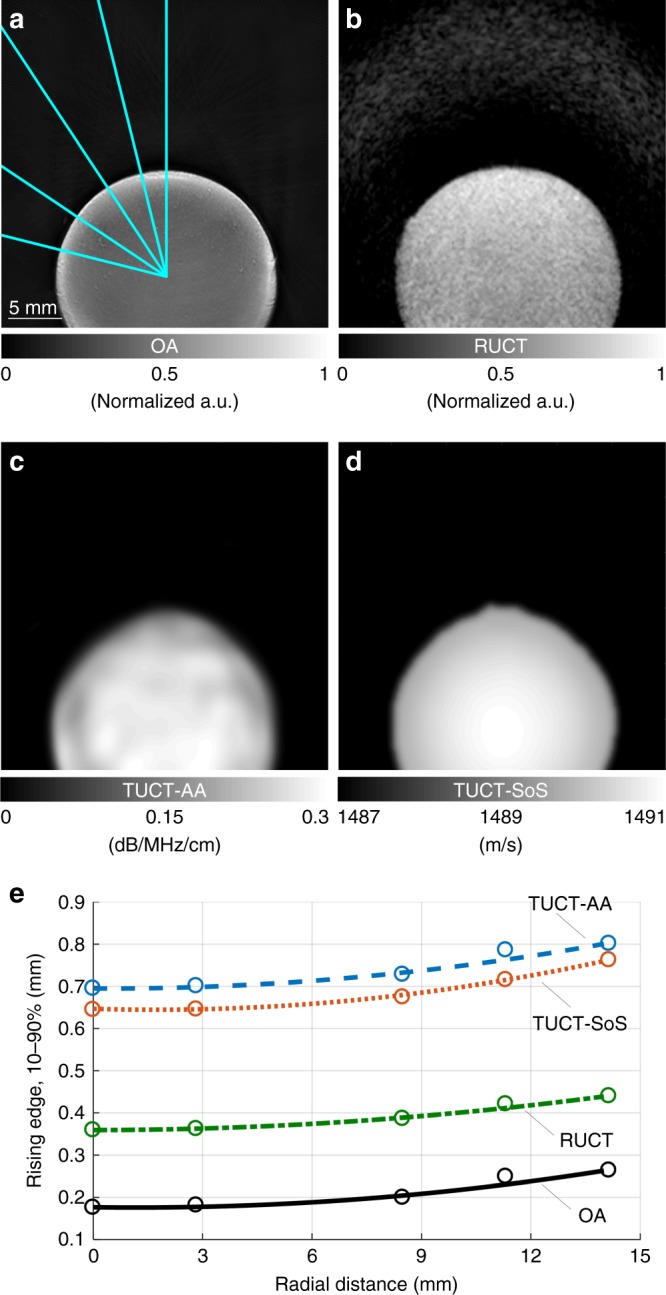
Imaging performance characterization of the TROPUS system in the tissue-mimicking phantom. **a** Optoacoustic tomographic reconstruction of the phantom. Trajectories of the radial intensity profiles used for the spatial resolution characterization are marked in blue. **b** The corresponding reflection ultrasound-computed tomography (RUCT) image of the phantom. **c** Acoustic attenuation image of the phantom reconstructed using transmission ultrasound (TUCT-AA). **d** The corresponding speed of sound (TUCT-SoS) image. **e** Measured and fitted in-plane resolution as a function of distance from the array center for all the four cases

Figure 3 displays the resulting multimodal images of the phantom. The mean and standard deviation of the pixel intensities in the circular region of interest (ROI) of 2-mm diameter in the phantom’s center were then evaluated for both the AA and SoS maps (Table 1). The latter could be recovered with a high accuracy with the deviation between the reconstructed and expected values of only 7.7% and 0.06%, respectively. To accurately characterize the spatial resolution across the imaging plane for all the supported imaging modes, the phantom was positioned with its edge crossing the center of the circular array geometry and the radial signal profiles were assessed along the trajectories indicated in Fig. 3a. The estimated in-plane spatial resolutions, shown as a function of the radial distance from array’s center, are displayed in Fig. 3e. The resolution values for both the OA and reflection ultrasound-computed tomography (RUCT) modes are comparable with those previously reported for a similar concave array with 270° angular coverage^5^. The spatial resolution in TUCT for both the AA and SoS modes is lower than for the other modes, but still within the submillimeter range. This is in agreement with what was previously achieved for Ray-theory-based TUCT reconstructions, where resolution in the order of 2–3 mm has been reported^38^.

**Table I:** Expected and measured values for the average speed of sound (SoS) and acoustic attenuation (AA) in the tissue-mimicking phantom

Property	Expected	Measured ROI
Acoustic attenuation [dB/MHz/cm]	0.26	0.28 ± 0.008
Speed of sound [m/s]	1492.1	1493 ± 0.04

### Whole-body mouse imaging in vivo

**Fig. 4:**
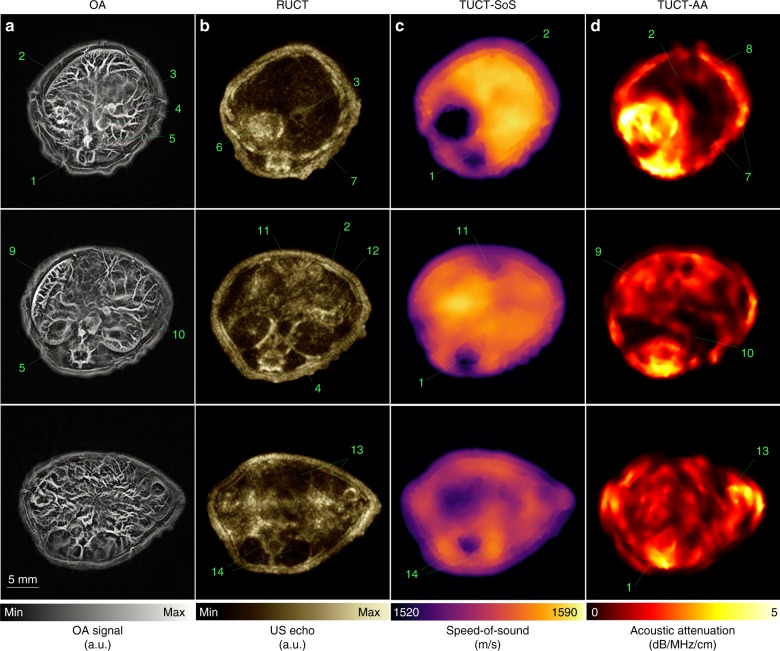
Hybrid transmission–reflection optoacoustic ultrasound (TROPUS) whole-body imaging. **a** Representative cross-sections acquired in the optoacoustic mode. **b** The corresponding reflection-mode ultrasound images. **c**, **d** The corresponding transmission-mode ultrasound images showing the distribution of the speed of sound and acoustic attenuation, respectively. Annotations: 1: spinal cord; 2: liver; 3: vena porta; 4: vena cava; 5: aorta; 6: stomach; 7: ribs; 8: skin/fat layer; 9: spleen; 10: right kidney; 11: cecum; 12: pancreas; 13: intestines; 14: muscle

The in vivo applicability of the developed TROPUS system was demonstrated via noninvasive whole-body imaging of a mouse. The animal was positioned in the upright position inside the imaging chamber (Fig. 1a) and maintained under anesthesia throughout the experiment. Representative cross-sectional reconstructions from the anterior to posterior regions of the mouse are presented in Fig. 4 for all the imaging modes. The fully coregistered images represent very diverse types of tissue contrast, thus reveal distinct and complementary anatomical and physiological information.

Since the OA contrast stems from optical absorption by tissue chromophores, most importantly oxy- and deoxyhemoglobin^39^, vessels and vascularized organs, e.g., the kidney or spleen, are clearly visible in the images. At the same time, large vessels, such as the thoracic aorta and vena cava appear anechoic (dark) in the RUCT images. Bones and skin provide clear hyperechoic (bright) contrast, while some organs, including the stomach and pancreas, produce more diffuse reflections leading to hypoechoic (gray-colored) contrast^5^. Additionally, considering that the US contrast stems from the acoustic impedance mismatch between different tissues, the RUCT images allow for an easy identification and boundary delineation of distinct organs.

The TUCT images, representing distribution of the SoS and AA across the mouse, provide complementary information to the tissue optical absorption and acoustic reflectivity delivered by the OA and RUCT modalities. The SoS depends on medium’s density and stiffness, both can be altered under certain disease conditions^40,41^. As expected, SoS fluctuations amount to ~5% in the healthy soft tissues (Fig. 4c). Missing transmitted acoustic signals create more significant SoS heterogeneities in bones as well as areas with air accumulation, such as lungs, stomach, or intestines. Absence of an alternative (reference) method for in vivo, SoS characterization hinders univocal validation of the reconstructed SoS values. However, those closely resemble the typical average values reported for soft tissues (1540 m/s)^42^, the liver (1573 m/s), or kidneys (1565 m/s)^43^. The reconstructed AA maps exhibit larger fluctuations^44^ of more than 60% in the average AA between the three cross-sections shown in Fig. 4d.

### Image quality enhancement

**Fig. 5:**
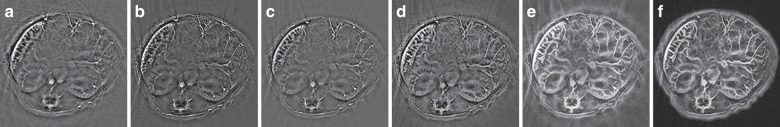
Enhancement of the optoacoustic image contrast. **a** Cross-sectional image of the mouse in the kidney area that was reconstructed using two-dimensional filtered back-projection algorithm assuming a uniform average speed of sound of 1542 m/s. **b** The same image reconstructed using a weighted back-projection algorithm. **c** Weighted back-projection reconstruction assuming different speed of sound of 1565 m/s for the mouse body and 1520 m/s in water surrounding the mouse. **d** Contrast enhancement with adaptive histogram equalization. **e** Application of the vesselness filter enhances contrast from vascular structures. **f** Background intensity suppression further enhances the image contrast

Raw optoacoustic images reconstructed using commonly employed filtered back-projection schemes are often afflicted by multiple artifacts, and thus, they may exhibit low contrast and insufficient level of anatomical detail (Fig. 5a). Loss of resolution associated with acoustic heterogeneities and uneven light deposition across the mouse can be partially mitigated by means of a statistically weighted back-projection algorithm^45^, resulting in fewer artifacts, clearer skin boundaries, and a finer detail of internal structures (Fig. 5b). Image resolution can be further improved and artifacts manifested via distorted shape of the peripheral vessels can be reduced by employing reconstruction that uses different speeds of sound inside the mouse and the surrounding water (Fig. 5c). Visual quality of the OA images is commonly impaired by the presence of highly absorbing vascular features and highly heterogenous light distribution in the object that lead to erroneous weighting of the recorded signals during the reconstruction process and appearance of hot spots in the images. A number of processing steps can be employed to improve the perceived image contrast. First, we applied contrast-limited adaptive histogram equalization (CLAHE) to enhance an overall image contrast (Fig. 5d). Contrast of the vascular structures can be further improved by using vessel enhancement filters^46–48^, as shown in Fig. 5e. This particular multiscale filtering approach, which employs Gaussian kernels of various sizes, can efficiently preserve and highlight blood vessels of different orientation and size^49^ while further emphasizing the mouse boundary. The final image enhancement step consisted of suppressing the background signals outside the mouse, thus further improving the perceived image quality (Fig. 5f). The newly developed full-view array geometry has greatly contributed to the overall visibility and resolution of anatomical structures across entire mouse cross-sections, thus mitigating artifacts that are commonly present in optoacoustic images acquired with limited-view tomographic systems^41,50^. However, the developed cross-sectional imaging system uses in-plane cylindrical focusing, which may result in loss of image quality due to anisotropic resolution in the vertical (*z*) versus lateral (*x–y*) dimensions.

## Discussion

In this work, we report on a new small animal imaging platform, termed transmission–reflection optoacoustic ultrasound (TROPUS), for concomitant noninvasive mapping in whole live mice of the absorbed optical energy, acoustic reflectivity, speed of sound, and acoustic attenuation with submillimeter resolution, further revealing the synergistic and complementary value of the newly developed multimodal combination. In vivo mouse imaging experiments revealed fine details on the organ parenchyma, vascularization, tissue reflectivity, density, and stiffness.

The diverse contrasts and superior imaging performance attained by TROPUS is of value for probing and quantification of multiple anatomical, functional, and molecular properties in health and disease. For instance, speed of sound and acoustic attenuation are known to be altered by breast malignancies and other neoplastic lesions^51^. Pulse-echo US is also an accepted method for detecting changes in elastic properties in several types of tumors^52–54^. Both RUCT and OA enable imaging at high frame rates and thus can render dynamic functional information, such as blood flow and distribution of oxygenated and deoxygenated hemoglobin^2,55,56^. Strong light absorption by melanin can be exploited to optoacoustically characterize skin lesions, circulating metastatic cells, or lymph node metastases^57^. The use of contrast agents and genetic reporters^58–60^ can further enhance the versatility and molecular sensitivity of the multimodal approach, potentially enabling new labeling and early disease detection approaches.

The information retrieved by one modality can be included as a prior knowledge to enhance reconstruction quality of the other modalities. Here, we used the SoS values obtained with TUCT in a two-compartment model for improving the OA reconstructions. Going forward, incorporating the full map of heterogeneous speed of sound distribution may yield further improvements in the OA image quality^61,62^. The AA and reflectivity maps can further be used for boosting the spatial resolution^35,63^ and removing artifacts^64^ in the OA images. Indeed, it was observed that the statistical-weighting-based reconstruction approach could mitigate artifacts associated with acoustic heterogeneities and uneven light deposition in the object^45^. The acoustically mismatched regions identified in RUCT images may potentially enable accurate modeling of scattering, refraction, and other US propagation effects as part of a full-wave inversion (FWI) scheme to overcome the relatively low resolution achieved with bend-ray tracing methods in TUCT^65^. Finally, the US and OA data can be combined to allow for a more accurate localization of acoustic sources^66,67^ and may also be used to mitigate the artifacts in the SoS maps for the bone and air regions. In the present work, the exclusion of reflections in the TUCT-AA reconstructions has resulted in the edges between tissues exhibiting higher values, as they represent the combination of absorption and reflection in that region, similarly to the so-called “edge-enhancement” in phase-contrast X-ray CT^68^. In future work, we aim to improve the TUCT algorithm by incorporating the full reflectivity information into the transmission reconstruction framework.

Beyond the small animal imaging domain, the TROPUS approach is of great interest for clinical translation. For one, integration of the well-established pulse-echo US imaging capability can facilitate clinical acceptance of the OA and TUCT methods. Recently, pilot clinical trials have demonstrated great value of the hybrid pulse-echo US and OA approach for the diagnosis of breast and skin cancer^9,69^ as well as inflammatory bowel (Crohn's) disease^6^. Likewise, TUCT has shown great promise in the breast cancer diagnostics and screening applications^70,71^.

In conclusion, the newly developed synergistic multimodal combination offers unmatched capabilities for imaging diverse tissue properties and biomarkers with high resolution, penetration, and contrast.

## Materials and methods

### Experimental system

The experimental setup for the hybrid transmission–reflection optoacoustic ultrasound (TROPUS) system is schematically depicted in Fig. 1a. The system comprises a dedicated multiplexer unit configured to control the custom-made full-ring US transducer array in two different operation modes in a time-interleaved fashion. In the receive-only mode OA signals are collected, whereas in the transmit-and-receive mode, the array was actively driven to generate US waves and acquire the reflected and transmitted signals.

The custom-made ring-shaped detector array of cylindrically focused transducers (Imasonic SaS, Voray, France) was designed to cover nearly 360° around the imaged object, which facilitates optimal data acquisition for transmission and reflection of US imaging as well as sufficient angular coverage for accurate OA tomographic imaging^41,72^. The array has 512 elements in total and consists of two concave subarrays, attached to each other by means of special locking mechanism, each having 256 elements, an active angular aperture of 174°, and radius of the curvature of 40 mm, with the individual elements focused at a distance of 38 mm. The modular design provides the flexibility to operate one of the 256-channel subarrays in a handheld mode. The elements have central frequency of 5 MHz, nominal Tx/Rx bandwidth of 60% and interelement pitch of 0.47 mm. For acquisition of 3D data in all the modes, the array was translated along the mouse using a vertically oriented translation stage (Model RCA2-TWA4NA, IAI Corporation, Shimizu-Ku, Japan).

OA signal excitation was done with a pulsed (~8 ns) Nd: YAG laser (Spectra-Physics, Santa Clara, CA, USA) with 10 Hz repetition rate and 1064 nm optical wavelength. An optical fiber bundle (LightGuideOptics GmbH, Rheinbach, Germany) composed of 620 fibers distributed over 12 output ferrules was fixed at both sides of the transducer array (Fig. 1a) at angular steps of 60°. The fibers in each output ferrule were arranged along a rectangular surface with dimensions 0.21 mm x 12.65 mm and oriented at ~24° angle with respect to the array’s imaging plane to achieve a uniformly illuminated ring with an area of ~6 cm^2^ on the surface of the mouse. The total per-pulse energy measured at the output of the fiber bundle was ~54 mJ, resulting in light fluence of 9 mJ/cm^2^ on the surface, which is well below the ANSI limit of 100 mJ/cm^2^ at 1064 nm^73^.

For collection of reflection- and transmission mode US data, the synthetic transmit aperture (STA) technique was employed (Fig. 1b) with active subapertures of 128 elements. Generation of a single-cycle bipolar pulse (20 Vpp, 6 MHz) from the first channel (TX#1) of the active subaperture results in an unfocused transmitted beam. The reflected (or back-scattered) US wave front is subsequently recorded by all the 128 channels of the active subaperture (RX#1–128). The active group is subsequently moved by one channel and the transmit–receive cycle is repeated for all channels, i.e., TX#2, RX#2–129; TX#3, RX#3–130;…TX#512, RX#512, 1–127. Transmission by the last channel (TX#512) and reception with the active group RX#512, 1–127 concludes the first acquisition cycle (AQ#1). Therefore, four acquisition cycles are required to collect reflected/transmitted signals for each pair of transmit–receive channels. The schematic matrix representation of the acquisition in Fig. 1c, with rows/columns corresponding to the transmit/receive (TX/RX) channels, shows the channels used in each of the four acquisition cycles. From this representation, one may observe how US data for any pair of transmit and receive elements were collected, e.g., for the 1^st^ transmit channel (TX#1), the echo waves were received by channels RX#1–128 in the 1st acquisition cycle (AQ#1), by channels RX#129–256 in the 2^nd^ acquisition cycle (AQ#2), and channels RX#257–384 and RX#385–512 in the 3^rd^ (AQ#3), and 4^th^ (AQ#4) acquisition cycles, respectively. Acquisition of reflection- and transmission US data was performed by a custom-built US data acquisition system (DAQ) having 128 transmit/receive channels (S-Sharp Corporation, Taiwan, China), 12-bit vertical resolution, 10 MHz input bandwidth, 24 MS/s sampling rate, and the function of triggered acquisition. In the implemented transmission ultrasound imaging scheme, a single element is excited for each transmission event with the driving voltage limited to 20 V. Unfocused transmit beams are generated in this way, thus imposing significantly lower levels of ultrasound exposure compared with conventional B-mode schemes using multielement transmission and focused transmit beams. In particular, the current FDA regulatory limit for adult diagnostic imaging is *I*_spta _= 720 mW/cm^2^ (spatial-peak-temporal-average)^74^, whereas in our case, the short-pulse transmission with a single element resulted in intensity levels well below 50 mW/cm^2,75^.

OA data acquisition was based on simultaneous collection of the generated signals by all the 512 channels of the array (Fig. 1c). Digitization was performed with a custom-made OA DAQ at 40 MS/s and 12-bit vertical resolution. The time required for OA tomographic data acquisition was ~50 µs, whereas ~86 ms were required for one full US image acquisition. The multiplexing unit synchronizing OA and US acquisitions was based on the predefined timing scheme triggered with the laser pulses (Fig. 1d).

### OA image reconstruction and postprocessing

For OA image reconstruction, the acquired signals were first preprocessed with a 3^rd^ order Butterworth bandpass filter (0.5 and 7 MHz cutoff frequencies) and deconvolved with the electrical impulse response of the transducer, simulated based on Krimholtz–Leedom–Matthaei (KLM) circuit model^76^ using the information provided by the manufacturer. Reconstruction was then performed with a statistical-weighting approach assuming different speed of sound in the sample and the surrounding water^64^. A value of 1520 m/s was assumed for water at 34 °C^77^. The mouse boundary was segmented from an OA image reconstructed assuming uniform speed of sound. The speed of sound value inside the mouse was assigned by considering the mean value of 95% top pixels of the reconstructed TUCT-SoS images. Specifically, values of 1573 m/s, 1565 m/s, and 1552 m/s were assumed for reconstructing cross-sections in the liver, kidney, and intestine regions of the mouse, respectively. A weighting-based back-projection algorithm was employed to mitigate image artifacts^45^ primarily stemming from the acoustic reflections from bones or air in areas, such as the spinal cord and intestine^78,79^. To improve signal-to-noise ratio (SNR) of the recorded OA signals, 50 frames were acquired for each position, out of which ~30% (15 frames) covering the breathing cycle were discarded, and the rest of the frames were averaged. For this, we employed a simple retrospective motion correction algorithm based on removal of frames with in-plane motion (2D)^4^. In particular, a similarity metric function is calculated for each of the selected cross-sectional (2D) slices, reflecting the level of similarity between a particular slice and the reference slice. The latter represents an artificial frame from the median of all PCA coefficients. Once all similarity metrics are calculated, a desired percentage of most deviating frames can be discarded.

To enhance contrast of the reconstructed OA images, several postprocessing steps were further applied. First, we used contrast-limited adaptive histogram equalization (CLAHE)^80^, with the Rayleigh distribution specified as a desired histogram shape. At the second step, vascular contrast was enhanced with the help of a multiscale vesselness filter^81^ applied at scales σ = 0.5, 1, 2, 3, 4. A two-dimensional vesselness filter was used instead of its 3D counterpart due to the relatively large step size between the adjacent slices. In addition, cylindrical focusing of the transducer array has resulted in an inferior spatial resolution in the elevation direction, thus making this particular tomographic configuration suboptimal for rendering the true 3D reconstructions required to accurately represent vascular structures in arbitrary directions^3^.

Note that the adaptive histogram equalization is applied to the image at each scale, followed by the weighted summation of the resulting images. At the final step, background suppression was applied to reduce nonzero background intensity. For this, the image was manually segmented into the foreground (mouse body) and background (surrounding coupling medium). The manual segmentation was performed under supervision of an experienced biologist and bioimaging expert by drawing polygons around the structures followed by spline interpolation of the polygon smoothening. The background intensity was then reduced by a factor of 1.25 compared with the intensity of the foreground.

### Reflection ultrasound-computed tomography (RUCT)

In the RUCT mode, a high-resolution image was created by coherent summation of the low-resolution images from 128 individual transmission events corresponding to one subaperture (SA). In particular, for each transmission event from a single element, echoes were collected by all the elements of the subaperture, and a low-resolution image was generated by means of a standard delay-and-sum algorithm^56^. The transmission is repeated for all the 128 subaperture channels. In the current study, a total of four 128-element subapertures were used to cover the complete 512-element aperture of the full-ring array. A final tomographic image was then generated with a spatial compounding technique^82^. This involves incoherent summation of the high-resolution images corresponding to different subapertures from multiple viewing angles. Transmit and receive channels that constitute the four subapertures (SA#1-4) used to compound a final image are shown in Fig. 1b. Beamforming was performed on the signal envelope extracted using the following preprocessing steps: baseband demodulation of the signals, low-pass filtering with cutoff frequency of 10 MHz to suppress noise, and upmixing to shift the frequency spectrum from the baseband back to its original band^83^. GPU-accelerated reconstruction of the RUCT images was performed on the US DAQ system and subsequently transferred as binary raw data files via Ethernet to the PC. The postprocessing steps for the final RUCT images included logarithmic compression, image upscaling by factor of 2 and conversion to RGB using a custom-built colormap “golden hue”^84^.

### Transmission ultrasound-computed tomography (TUCT)

In the TUCT mode, the transmission data were used to reconstruct SoS and AA maps. For this, the time-of-flight (TOF) and signal attenuation were determined for each emitter–receiver pair. The TOF was obtained following the method proposed elsewhere^85^. The TOF picker code from E. Kalkan^86^ was adapted to improve the accuracy of the selected TOF picks by weighting in the TOF values around the selected TOF, as described elsewhere^87^. No additional signal denoising was needed before the TOF picker^88^ due to the high SNR of the signals. Median filtering and reciprocal pair comparison were incorporated in the TOF picker to effectively remove the outliers^87^. Signal attenuation was determined using the complex signal energy ratio method^87^. The acoustic attenuation coefficients are related with the measured signals as follows:

1 $$\mathop {\sum}\nolimits_{j = 1}^n {{\kern 1pt} A_{i,j}\alpha _{0j} = \frac{1}{{f_c}}{\mathrm{ln}}\left( {\frac{{I_i^\prime }}{{I_i}}} \right)}$$

where *A*_*i*,*j*_ represent the propagating path along the ray determined by the emitter–receiver pair *i* within pixel *j*, $\alpha _{0j}$ is the attenuation coefficient at the pixel *j*, $I_i^\prime$ is the amplitude of the signal for the emitter–receiver pair *i* when there is only water in the field of view (FOV), *I*_*i*_ is the corresponding amplitude with the object located in the FOV, and *f*_*c*_ is the central frequency of the signal.

The TOF and signal attenuation values extracted from the transmitted data served as the input for a bent-ray-tracing iterative reconstruction algorithm^38^. In this work, the shortest (geodesic) path between each pair of ultrasound transducers for a given SoS map was modeled as a Bézier curve^89,90^. The geodesic path was selected among a family of quadratic Bézier curves connecting the emitter and the receiver as the curve providing the lowest TOF. Among various methods suggested for obtaining the geodesic path^38,91^, our approach has the advantage of exploiting the large capabilities of graphics processor units (GPU) to perform many computations in parallel. The TOF values along the different Bézier curves were evaluated in parallel using separate GPU threads. Once the geodesic paths have been determined, Maximum Likelihood–Expectation Maximization (ML-EM)^91^ was used to solve the optimization problem for the SoS and the AA maps as follows:

2 $$\xi _j^{(n + 1)} = \frac{{\xi _j^{(n)}}}{{\mathop {\sum}\nolimits_{i{\prime} = 1}^M {{\kern 1pt} A_{i{\prime},j}} }}{\kern 1pt} \mathop {\sum}\nolimits_{i = 1}^M {{\kern 1pt} A_{i,j}\frac{{p_i}}{{\mathop {\sum}\nolimits_{j{\prime} = 1}^N {{\kern 1pt} A_{i,j{\prime}}{\kern 1pt} \xi _{j{\prime}}^{(n)}} }}}$$

where $\xi _j^{(n + 1)}$ is either the speed of sound or the absorption coefficient value in the pixel *j* for iteration n + 1 based on the value $\xi _j^{(n)}$ for iteration n. *A*_*i,j*_ is the coefficient defined in Eq. (1), and *p*_*i*_ represents the measured pressure field at the receiver location *i*. A total of 50 iterations (image updates) with a one-step-late maximum a posteriori (MAP) regularization based on the median prior^92^ were used for reconstructing each 2D slice. For visualization, the mean intensity values of the background of the TUCT-SoS and TUCT-AA images were calculated and set as minimum threshold such that the intensity values lower 1520 m/s and 0.002 dB/MHz/cm for the TUCT-SoS and TUCT-AA images, respectively, were not displayed in the final RGB images.

All postprocessing steps were performed in MATLAB (2016b, MathWorks, Natick, MA, USA) on a desktop computer with Intel Core i7-4820 K 3.7-GHz processor and 32-GB RAM.

### Characterization of the imaging performance

A tissue-mimicking phantom with known speed of sound and acoustic attenuation was used to test the imaging performance of the hybrid system. The phantom had a cylindrical shape with diameter of 20.0 ± 0.2 mm and length of 50 ± 0.2 mm. Acoustic attenuation was mimicked with glass microspheres (Spheriglass A, Potter Industries LLC, Malvern, PA, USA) with diameters between 38 and 63 μm added to the agar-based solution. Table 2 summarizes the concentrations of each additive for the tissue-mimicking phantom.

**Table 2:** Tissue-mimicking phantom composition

Component	Concentration	Unit
Water	100	ml
Agar	1.3	g
Ink	0.2 (1:500 dilution)	ml
Intralipid	6	ml
Glass microspheres	2.5	g

Acoustic properties of the phantom material were characterized with an acoustic transmission test^93^. For this, a fiber bundle was coupled to the Nd: YAG laser, and a thin wire served as an optoacoustic source. The phantom was immersed in a water-filled container. A single-element transducer (Sonotec, Halle, Germany) with 10-mm element size, 5 MHz center frequency, and 80% bandwidth was used to collect the transmitted signals, and a photodiode was positioned near the output of the excitation laser source to trigger the oscilloscope acquisitions.

The OA signals generated by the wire were acquired for the container only filled with water as a reference and with the phantom material immersed. The speed of sound within the sample *c*_*s*_ was determined from the temporal shift Δ*t* between the position of the OA signal peak with and without the sample in the signal path, as follows:

3 $$c_s = \left( {\frac{1}{{c_w}} - \frac{{{\mathrm{\Delta }}t}}{d}} \right)^{ - 1}$$

where *c*_*w*_ is the speed of sound in water [m/s], *d* is the sample thickness [m], and Δ*t* is the time shift of the signal peak with the sample in place relative to the reference signal in water [s]. The speed of sound in water was calculated according to the formula for the sound speed in distilled water as a function of temperature^77^. The effect of temperature on the speed of sound of the material was estimated with measurements conducted at 21 °C, 23 °C, and 25 °C. The target temperature was achieved with an electronic submersible water heater (Model 560, Offenbach, Schego GmbH, Germany) regulated with a thermostat. Three measurements were conducted three times - at 5, 10, and 15 min, to ensure the temperature stability and the measured values were  fitted with a 2^nd^ order polynomial using least squares method.

The frequency dependence of the acoustic attenuation was calculated from the log difference between the retrieved spectra as follows:

4 $$\alpha (f) = - \frac{{20}}{{d \cdot 10^2}}{\it{log}}_{10}\frac{{A_s(f)}}{{A_w(f)}}[{\mathrm{dB}} \cdot {\mathrm{cm}}^{ - 1}]$$

where *A*_*s*_(*f*) was the magnitude of the spectrum with the sample in place, and *A*_*w*_(*f*) was the magnitude of the spectrum with no sample in place. The measurements were conducted three times, and the measured values were fitted by the frequency power law as follows:

5 $$\alpha (f) = \alpha f^b$$

The tissue-mimicking phantom was further used to characterize the resolution in the OA, RUCT, and TUCT modes. For this, the transducer array was positioned relative to the phantom, with the center of the cylinder having coordinates (*x*, *y*) = (0, 10) [mm], and its edge was located at the center of the circular transducer array geometry, i.e., at (*x*, *y*) = (0, 0) [mm]. The resolution of the system as a function of distance from the array center was estimated by analyzing the different radial intensity profiles. Specifically, the resolution was calculated as the edge response defined as the distance in the image between the points having intensities of 10 and 90% from the maximum image value^94^.

### Animal imaging

A healthy ICR (Imprinting Control Region) albino mouse was used for in vivo imaging. The animal was anesthetized with a mixture of 1.8% isoflurane in 100% oxygen at a 0.8 L/min flow rate, positioned into the imaging chamber in the upright position using a custom-designed animal holder, and maintained under anesthesia throughout the experiment. The water temperature was strictly maintained at 34 °C with an electric heater (Model 560, Offenbach, Schego GmbH, Germany). A series of cross-sectional images were subsequently acquired from the anterior to posterior regions with a 2-mm step size. All procedures involving animal care and experimentation were conducted in full compliance with the institutional guidelines of the Institute for Biological and Medical Imaging and with approval from the government of Upper Bavaria.
